# The impact of cultural understanding-based approach to teaching Chinese characters on primary school students' performance, motivation, cognitive load, and culture understanding

**DOI:** 10.3389/fpsyg.2026.1839204

**Published:** 2026-07-21

**Authors:** Yulu Jin, Baowen Shao, Nik Muhammad Hanis, Gang Yang, Haiying Qiu

**Affiliations:** 1 Faculty of Human Development, Sultan Idris Education University, Tanjong Malim, Perak, Malaysia; 2 School of Marxism, Shandong Huayu University of Technology, Dezhou, Shandong, China; 3 Department of Educational Technology, College of Education, Wenzhou University, Wenzhou, China; 4 Shimen Town Centre Primary School, Tongxiang City, Jiaxing, China

**Keywords:** cognitive load, cultural understanding, learning motivation, primary education, teaching Chinese characters

## Abstract

Chinese characters are a core component of the Chinese language and a carrier of cultural connotations, making them vital in primary Chinese language education. However, the Traditional Stroke-by-Stroke Approach to Teaching Chinese Characters (TSSA-TCC) often fails to engage students or foster cultural understanding and identity. In order to enhance the implementation of Chinese character instruction, this study proposes a Cultural Understanding-based Approach to Teaching Chinese Characters (CUA-TCC). This study adopted a quasi-experimental pre-test–post-test control group design to investigate the effects of CUA-TCC on primary school students' performance, motivation, cognitive load, and cultural understanding. Participants comprised 77 s-grade students aged 8–10 years, who were divided into an experimental group (*n* = 38; 18 girls and 20 boys) and a control group (*n* = 39; 18 girls and 21 boys). The collected data were analyzed using a mixed-methods approach. Quantitative data were analyzed using independent-samples *t*-tests, while qualitative interview data were transcribed, coded, and analyzed thematically with the support of word cloud visualization. The study results showed that CUA-TCC not only enhances students' literacy skills and learning motivation and reduces cognitive load but also promotes the development of students' cultural understanding and national identity. Therefore, this study concluded that cultural elements should be an important part of Chinese character instruction. Meanwhile, the conclusions of this study provide theoretical support and practical insights for Chinese language education in primary schools and contribute to the field of teaching Chinese characters.

## 1 Introduction

Chinese characters are a core component of the Chinese language and one of the oldest scripts in the world, which is an important carrier and distinctive symbol of Chinese culture. The unique nature of Chinese characters with orthographic, phonetic, semantic, and grammatical information makes it impossible to replace them with the phonetic system alone (Jin, 2019). Moreover, it carries rich cultural connotations with its unique ideographic system and complex structure (Chen, 2023). Chinese character learning is crucial in Chinese language education. Especially in the low grade, which is a critical stage of language development, and the development of literacy skills has a crucial impact on reading comprehension, writing skills, and overall subject performance (Wickramasinghe et al., 2024), making Chinese character instruction essential in primary education. Literacy approaches are a series of strategies to enhance learners' literacy skills, which focus on the memorization and recognition of characters' phonetics, orthographic features, and structures (Chartier, 2016). Common approaches in Chinese character instruction include the phonetic approach, orthographic approach, contextual approach, component-based approach, and etymological approach (Fu and Tan, 2023; Hong and Huang, 2013). The etymological approach is the rationale for character construction, by linking the orthographic, phonetics, and semantics of a character (Zhu and Shen, 2022). However, its practical application often emphasizes character orthographic forms while neglecting cultural aspects, leading to superficial understanding and preventing students from fully grasping their cultural connotations (Pan and Song, 2024). Other common traditional approaches, such as Traditional Stroke-by-Stroke Approach to Teaching Chinese Characters (TSSA-TCC), emphasize stroke-order learning and repeated handwriting practice (Hsiung et al., 2017). Such approaches reinforce basic literacy but make writing practice boring and less effective (Christiana, 2013). It may fail to develop deeper cultural insights and identities or to help students understand the wider meanings of linguistic symbols within a particular cultural framework (Kristiawan et al., 2022).

Language and culture are inseparable and complementary to each other. Culture is the context and carrier of language, at the same time, language can reflect and contribute to the formation of culture (Krasniqi, 2019). Together, they construct the framework for human communication and cognition. Therefore, cultural learning is an integral part of language acquisition (Han, 2019). Furstenberg and English (2016) highlight that cultural understanding is a core component of language learning, which can enhance students' cultural literacy and language proficiency through effective teaching approaches. Moreover, the Ministry of Education of the People's Republic of China (2024) has proposed in its latest policy report that students should be guided to understand the historical and cultural values of Chinese characters while developing their character writing skills, to establish a sense of cultural identity. This policy confirms the importance attached by academics to the integration of language and culture in education. Therefore, beyond mastering linguistic symbols, learners need to develop a deeper understanding of culture (Hossain, 2024).

Therefore, there is a need to incorporate cultural elements into literacy education to develop students' language comprehension. In recent years, researchers have proposed integrating cultural elements into literacy instruction, for example, by telling the origin and cultural background of words to enhance students' dual knowledge of language and culture (Kristiawan et al., 2022; Purnamasari and Wahyuni, 2024). According to Purnamasari and Wahyuni (2024), integrating cultural contexts helps students establish meaningful connections and adopt an identity-centered approach to literacy that can improve literacy skills, learning motivation, and classroom engagement. However, traditional approaches may not adequately address this issue. Kristiawan et al. (2022) found that integrating local cultures into the learning of the mother tongue helped in better understanding and retention of vocabulary and concepts, fostered students' pride in their own identities and sense of cultural identity, and strengthened their motivation to learn. Zhu and Shen (2022) argue that the origin, development, and stories of Chinese characters should be explained based on an etymological approach, especially pictographic characters.

Such literacy approaches based on cultural understanding not only promote a deeper understanding of written symbols, and inspire greater learning interest and motivation, but also provide a richer learning experience and foster students' cultural confidence. This is because they associate linguistic symbols with cultural narratives and values. This promotes patriotism, cultural diversity, and national identity, thus instilling core values to counteract the loss of traditional practices in the process of globalization (Riyanto et al., 2024).

Based on the above analysis and thinking, this study aims to investigate the effects of Cultural Understanding-based Approach to Teaching Chinese Characters (CUA-TCC) on students, to assess its effectiveness in terms of students' performance, motivation, and cognitive load, and to examine its potential role in cultural awareness. This study examines the teaching of Chinese characters from a cultural perspective and analyses the results of CUA-TCC in comparison with TSSA-TCC. To provide new insights into the study of Chinese character education and to explore effective strategies for enhancing cultural identity while improving literacy skills. The research questions are as follows:

1. Compared with TSSA-TCC, does CUA-TCC more effectively improve students' performance in Chinese characters?
2. Compared with TSSA-TCC, does CUA-TCC more effectively increase students' motivation?
3. Compared with TSSA-TCC, does CUA-TCC more effectively reduce students' cognitive load?
4. Compared with TSSA-TCC, does CUA-TCC more effectively promote students' cultural understanding of Chinese characters?

## 2 Literature review

### 2.1 Culture and language learning

Cultural context plays a key role in language instruction, particularly through the use of cultural stories. Studies have shown that cultural stories, by visualizing the language learning context, enable students not only to learn the language itself and improve their literacy but also to gain a deeper understanding of the cultural context and emotions underlying the words (Obidovna and Tokhirovna, 2024). For instance, Takahashi (2010) emphasized the need to understand cultural norms, such as Japanese politeness strategies, which can enrich language learning and contribute to the comprehension and retention of linguistic features. These studies indicate that cultural stories enhance linguistic comprehension and retention by contextualizing language within cultural expectations and communication norms. Snow et al. (2020) found that Aboriginal storytelling by elders fostered identity comprehension and literacy engagement. Similarly, Cataldo and Alanís (2021) further demonstrated that cultural practices, such as using family portraits, significantly enhanced early literacy skills among Latino children, highlighting the critical role of cultural experiences in their literacy development. Moreover, Oktarina et al. (2022) found that integrating cultural content significantly increased students' learning motivation and comprehension. Thus, the integration of historical stories, traditional practices, and native cultural elements into the language learning process not only increased learners' engagement and retention in language learning but also enhanced students' interest in learning and cultural identity on an affective level.

### 2.2 Chinese character culture and Chinese character learning

Integrating cultural elements into primary school Chinese character instruction is crucial. Chinese scholars have conducted in-depth studies on how Chinese character culture works in literacy education. Zheng and Su (2018), based on the current situation of literacy instruction, proposed three macro-strategies: innovating teaching methods, expanding school-based Chinese character culture curriculum, and investing in teaching materials. Li (2020) further emphasized that cultural integration can help students perceive the profound Chinese culture, and enhance their cultural confidence, learning interest, and independent literacy skills. Preliminary international research supports this view. Christiana (2013) conducted an experiment with 18 Indonesian students, finding that incorporating cultural context in Chinese character instruction significantly improved students' learning motivation and effectively enhanced their Chinese character writing skills. He argued that the traditional teaching approach (TSSA-TCC) was often dull, while the cultural context made learning more attractive, engaging, and effective with vivid historical and cultural connotations. This provides valuable insights and inspiration for the present study. However, despite existing theoretical strategies, empirical research on their practical implementation remains limited. This study aims to bridge this gap by examining the effectiveness of cultural integration in Chinese character instruction.

### 2.3 Culture and learning motivation

The incorporation of cultural elements plays a crucial role in shaping learning motivation. There are various theories explaining learner motivation, with specific theories such as Attribution Theory suggesting that culture influences learner's motivation and its correspondence with learning outcomes (Ogan et al., 2009). According to Heine (2007) and Markus (2016), contextual and structural aspects of culture can create availability that affects motivation. In collectivist cultures, such as those in many Asian countries, group harmony, and community goals are often emphasized, while the self is often seen as interdependent with others. This cultural perspective motivates individuals to strive for the success of the group and inspires motivation for behaviors that promote group cohesion and support (Heine, 2007). Lo (2024) study further highlighted that cultural factors can influence students' learning motivation by fostering self-directed learning and deeper content engagement. Obidovna and Tokhirovna (2024) demonstrated that cultural storytelling effectively enhances students' learning motivation by connecting them to their identity, capturing attention, and stimulating interest. This approach encourages greater participation in literacy practices while fostering self-esteem and confidence, leading to more active self-expression. Handayan et al. (2024) further highlighted that engaging in cultural storytelling within meaningful contexts facilitates vocabulary acquisition and language development.

### 2.4 Cultural and cognitive load

From a cognitive perspective, cultural stories provide multidimensional meanings and contexts for learning content (Bowman, 2018), which can reduce the cognitive load to some extent (Gatti and Hoffmann, 2024). Ghafar (2024) found that cultural stories allow learners to emotionally and cognitively connect with the learning material by promoting active participation, which facilitates memory retention. A study of Bugis cultural narratives showed that children exposed to culturally relevant storybooks demonstrated significant cognitive improvements, with scores rising from 11.06 to 13.19 (*p* < 0.001) (Andi et al., 2024). These narratives not only develop cognitive skills but also instill cultural values and enhance overall cognitive engagement. However, the complexity of language in cultural stories can increase cognitive load, as higher language demands may slow reaction times and increase cognitive effort, impacting test performance and comprehension (Cormier et al., 2014), while variations in accents can further influence visual cognition and processing speed (Borges, 2014). Thus, different researchers have different views on how to balance the cognitive load in terms of culture. This needs to be further argued.

## 3 Methodology

### 3.1 Research design

Quasi-experimental research design was adopted to examine the impact of CUA-TCC and TSSA-TCC on Chinese character learning, motivation, and cognitive load. The experimental group used CUA-TCC, incorporating cultural background, origin stories, and pictographic features of Chinese characters. In contrast, the control group used TSSA-TCC, which emphasized character pronunciation, stroke order, repeated writing practice, and textbook-based exercises. Basic radicals and character components were introduced to support character recognition and writing, but instruction focused primarily on their linguistic functions rather than their cultural or historical meanings. By comparing both groups under different teaching approaches, this study explores the role of cultural context in Chinese character learning.

### 3.2 Participants

This study was conducted in spring 2024 at a public primary school in eastern China with second-grade students (aged 8-10). The experimental group included 38 students (18 girls and 20 boys), and the control group had 39 students (18 girls and 21 boys). After random sampling, pre-test results showed no significant difference in literacy skills between groups (*p* = 0.703 >0.05). Participation was voluntary with informed consent from parents, the school, and teachers. Both groups were taught by the same teacher using the same instructional materials, learning objectives, teaching duration, and assessment instruments, differing only in the teaching approach.

### 3.3. Learning materials

The learning materials were from the second-grade Chinese language textbook of the People's Education Press of China, focusing on the literacy unit with the theme “I am Chinese.” The cultural goals were divided into four parts: perceiving the magic of Chinese characters, understanding the motherland's landscapes, learning about traditional Chinese culture, and appreciating Chinese food culture. Both groups learned the same content, but the instructional approaches differed. The experimental group incorporated cultural stories and character evolution before Chinese character writing practice, while the control group used traditional glyph splitting without cultural context. For example, the character “贝” (meaning “shell”) was taught as follows:

#### 3.3.1 Morphological evolution of shell (“贝”)

**Figure 1:**
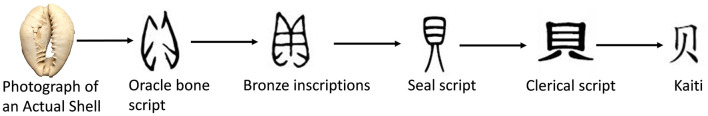
Morphological evolution of the Chinese character shell.

The character “贝” evolved from the original physical drawing of a shell and gradually changed the writing of oracle bone script, gold inscriptions, seal script, clerical scripts, and Kaiti (See Figure 1).

#### 3.3.2 Cultural significance of shell (“贝”)

Firstly, the Chinese character for shell (“贝”) originated from oracle bone inscriptions, resembling an open shell. In ancient China, people regarded shells as beautiful and precious and often used them as ornaments. Therefore, shell (“贝”) is used to describe objects shaped like shells, such as shell (“贝壳”), scallop (“扇贝”), and even figuratively in words like treasured or baby (“宝贝”).

Then, as history progressed, shells served as currency for trading because they were small, delicate, unbreakable, and easy to carry and count. Though later replaced by metal and paper money, shell (“贝”) retained its association with wealth and trade, appearing as a semantic component in characters like wealth (“财”), goods (“货”), expensive (“贵”), and cheap (“贱”).

### 3.4 Instruments

#### 3.4.1 Chinese character learning performance scale

The learning performance scale was developed regarding the Compulsory Education Chinese Language Curriculum Standards Ministry of Education of the People's Republic of China, 2022. It consists of 13 main items measuring four aspects: Chinese character recognition, writing, semantic understanding, and cultural cognition. Recognition tested students' ability to identify Chinese characters, distinguish character forms, and recognize pronunciations. Writing tested students' ability to accurately produce Chinese characters according to pinyin, contextual cues, and sentence construction tasks. Semantic understanding tested students' ability to comprehend character meanings and interpret language use in context. Cultural cognition tested students' understanding of culturally embedded knowledge represented in Chinese characters and texts.

Some items involve multiple dimensions (e.g., recognition and writing, writing and semantic understanding, or semantic understanding and cultural awareness) and therefore exhibit a degree of cross-dimensionality. In order to reflect the intrinsic links between various abilities in the process of learning Chinese characters, this study did not strictly categorize these items into a single dimension, but rather allowed them to correspond to multiple learning objectives during the test design phase. These four dimensions primarily serve as the theoretical framework for test development, ensuring that the test content covers all aspects of Chinese character learning; statistical analysis uses the overall score as an indicator of students' performance in Chinese character learning.

The test includes a picture-based writing task, which is assessed subjectively. To ensure consistency in assessment, this task is evaluated by a trained scorer using a predefined scoring rubric. The remaining items were scored against standard answers.

The total score ranged from 0 to 100. The same test was administered as both the pre-test and post-test to evaluate changes in students' Chinese character learning performance over the intervention period. The pre-test and post-test scores showed a strong positive correlation (Pearson's *r* = 0.897). To establish content validity, the instrument was evaluated by five experts in Chinese language education and educational measurement. Each expert independently rated the relevance of each item using a four-point scale (1 = not relevant, 2 = somewhat relevant, 3 = quite relevant, and 4 = highly relevant). The Scale-level Content Validity Index (S-CVI/Ave) was calculated as the average of the Item-level Content Validity Index (I-CVI) values across all items, yielding an S-CVI/Ave of 0.91, indicating good content validity.

#### 3.4.2 Learning motivation scale

This study refers to the existing motivation scale developed by Hwang et al. (2013). Several items were contextually modified by replacing general learning references with Chinese character learning contexts to better reflect the objectives of the present study. The revised scale consisted of 7 items across 2 dimensions —intrinsic motivation (3 items) and extrinsic motivation (4 items)—rated on a 5-point Likert scale. The Cronbach's alpha values for the two dimensions are 0.761 and 0.772, respectively, indicating good-scale reliability.

#### 3.4.3 Cognitive load scale

This study refers to the cognitive load scale of Paas (1992) and Sweller et al. (1998). Following the same adaptation procedures described for the Learning Motivation Scale, the items were revised to fit the Chinese character learning context. The scale consisted of 8 items across 2 dimensions: mental load (5 items) and mental effort (3 items), rated on a 5-point Likert scale. The Cronbach's alpha values were 0.88 and 0.77, respectively, indicating good reliability.

#### 3.4.4 Interview protocol

The design and preparation of the content of the interview outline for this study were based on the interview protocol of Fadzli et al. (2020), and Kvale (2009). It includes 4 aspects: interest in learning Chinese characters, comprehension of learning Chinese characters, cultural awareness, and cognitive load.

#### 3.4.5 Translation and adaptation of instruments

The Learning Motivation Scale and Cognitive Load Scale were originally developed in English and were translated into Chinese before administration. The translated versions were reviewed by bilingual researchers to ensure linguistic accuracy and conceptual equivalence. The final Chinese versions were administered to all participants. The interview protocol was also prepared in Chinese to ensure that the questions were age-appropriate and easily understood by the participating students.

#### 3.4.6 Data collection and analysis

Data collection includes quantitative and qualitative methods. Quantitative data are derived from the Learning Motivation Scale, Cognitive Load Scale, and Chinese Character Learning Performance Pre- and Post-tests to compare the experimental and control groups. Qualitative data come from audio-recorded, transcribed, and coded interviews. Combining both analyses offers a comprehensive understanding of the effects and potential benefits of culturally integrated Chinese character instruction.

### 3.5 Research procedure

**Figure 2:**
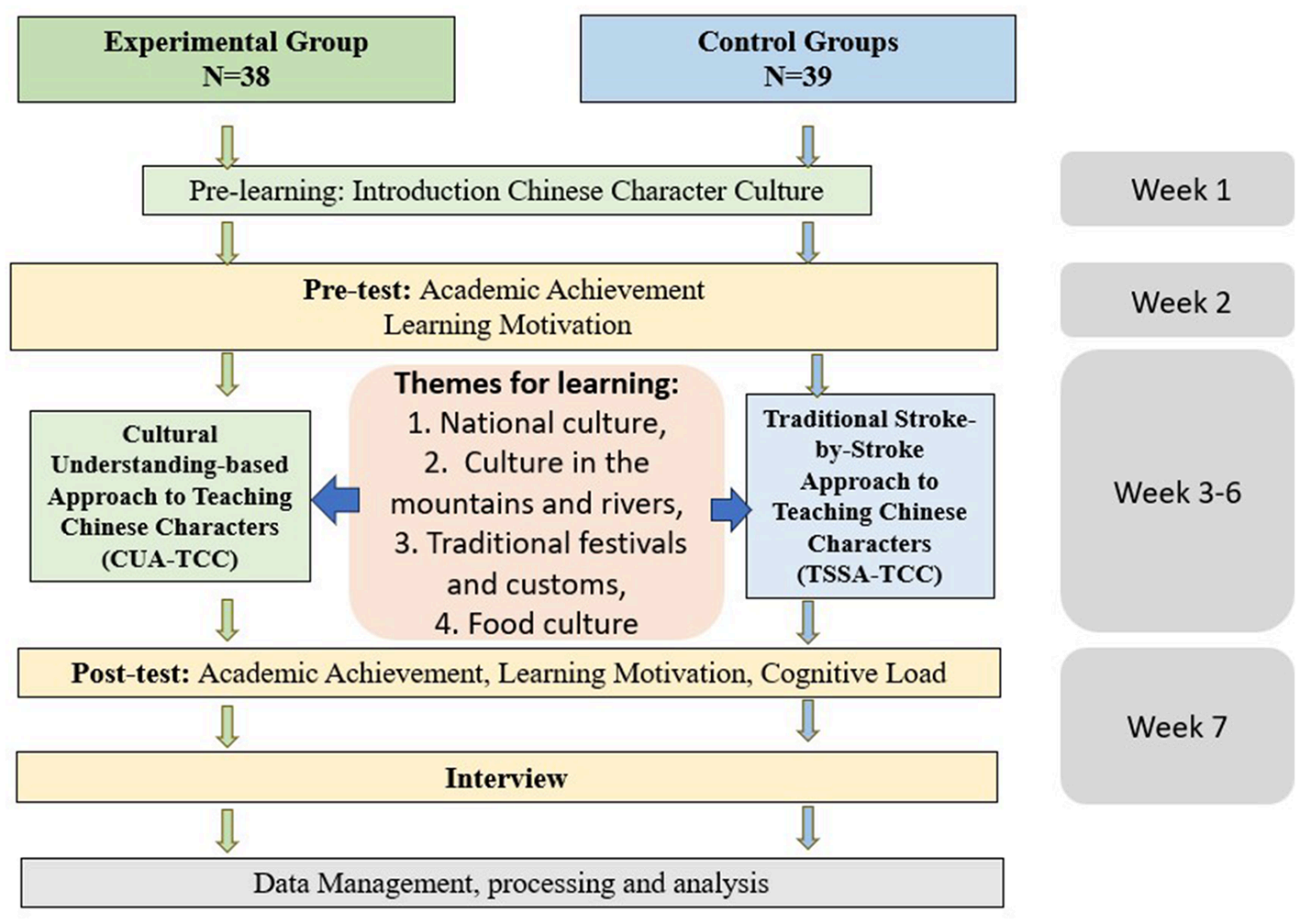
Experimental procedure over the 7-week study.

The 7-week experimental period consists of three phases: pre-test, intervention, and post-test (see Figure 2). In Week 1, both groups received a brief introduction to Chinese character culture. In Week 2, the pre-test instruments, including the Academic Achievement Test and Learning Motivation Scale, were administered to both groups prior to the intervention. The intervention was conducted from Week 3 to Week 6. During this period, the experimental group received instruction using the CUA-TCC, while the control group was taught using the TSSA-TCC. Both groups learned the same thematic content and followed the same teaching schedule. In Week 7, the post-test instruments, including the Academic Achievement Test, Learning Motivation Scale, and Cognitive Load Scale, were administered to both groups. In addition, semi-structured interviews were conducted with the participated teacher and selected students from both groups. Finally, the collected data were organized and analyzed.

### 3.6 Instructional process

#### 3.6.1 Instructional process of the experimental group

**Figure 3:**
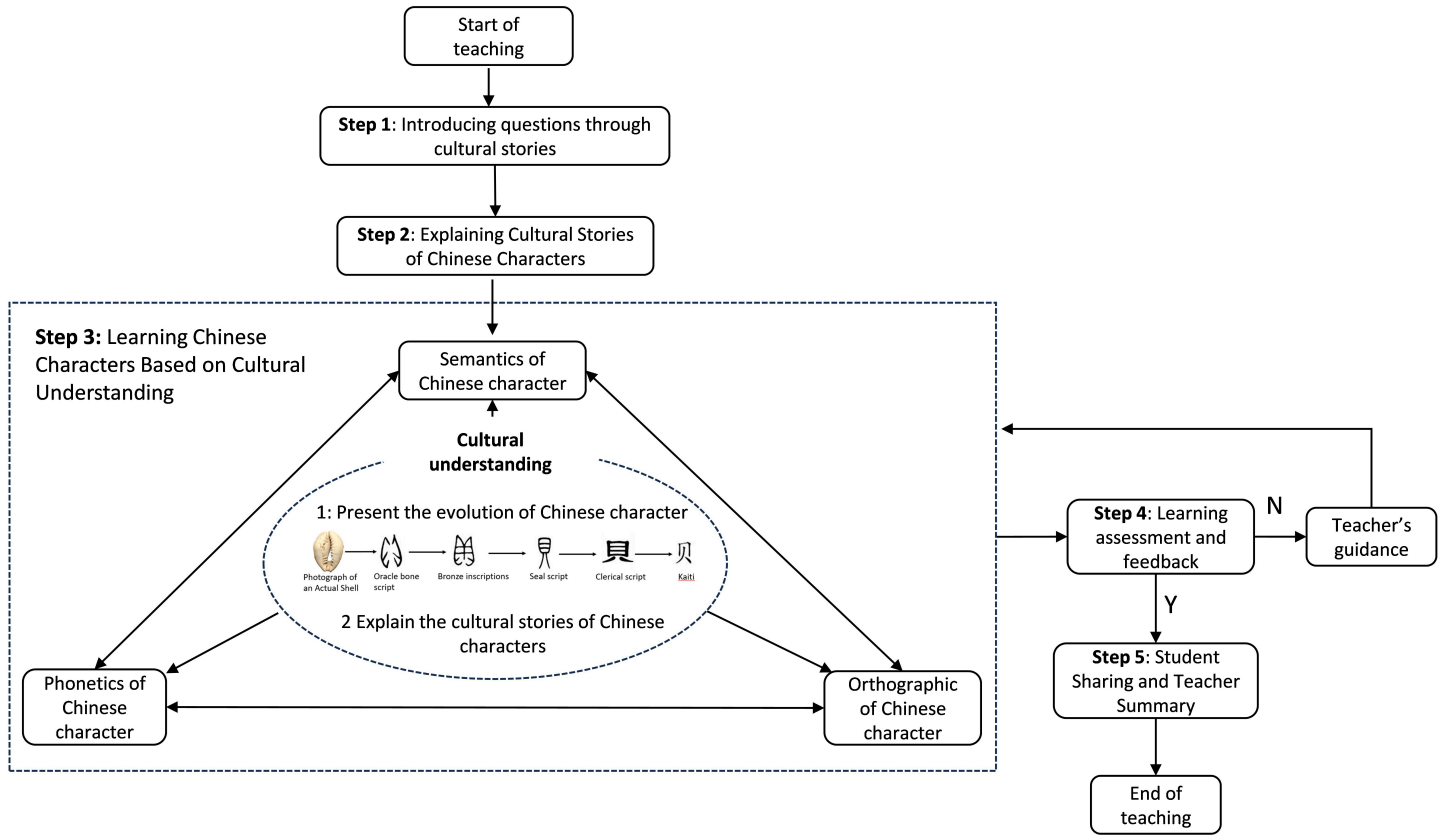
Flow chart of the instruction.

The instructional process of the experimental group consists of five key steps (Figure 3).

Step 1 involves thought-provoking questions to spark students' curiosity and connect the character to its cultural context. For example, teaching shell (“贝”): “In ancient times, people didn't use mobile phones or money. Can you guess what did people use for trade?”

Step 2 is to explain the historical and cultural significance of characters, providing context for deeper understanding and laying the foundation for subsequent instruction.

Step 3 is characters learning through cultural understanding. For semantics, cultural stories are used to illustrate the meaning of the character (e.g., the association of shell (“贝”) with wealth and trade). For orthographic forms, the evolution from oracle bone inscriptions to modern glyphs is demonstrated so that students can visualize the changes. For phonetics, students practice pronunciation in a cultural context.

Step 4 is learning assessment and feedback. Students need to complete exercises while the teacher observes, corrects mistakes, and offers guidance.

Step 5 is reflection and summary. Students share what they have learned, and the teacher closes the session, emphasizing key cultural elements.

#### 3.6.2 Instructional process of the control group

The control group was taught using the TSSA-TCC, an instructional approach that mainly focused on repetitive practice of character pronunciation, stroke order, and writing (Hsiung et al., 2017). First, the teacher introduced the pronunciation and meaning of the characters. Relevant, basic radicals and character components were also introduced to support students' understanding of character forms and meanings. Next, students learned the stroke order and practiced writing the characters repeatedly through copying exercises. The teacher then explained the usage of the characters and guided students to complete textbook-based exercises. Finally, students reviewed and memorized the characters through repetition and drill-based activities. Although radicals and components were introduced in the control group, instruction primarily focused on their linguistic functions rather than their cultural or historical significance. Unlike the experimental group, the control group did not receive cultural stories, historical explanations, or character evolution instruction during the learning process.

## 4 Results

### 4.1 The difference in chinese character learning performance

The pre-test results (Table 1) indicate no significant difference in Chinese Character learning performance between the two groups (*t* = −0.382, *p* = 0.703 > 0.05), indicating comparable initial levels before the experiment. However, post-test results (Table 2) show that the experimental group outperformed the control group in overall scores (ME = 91.84 > MC = 88.12), Chinese character recognition (ME = 34.12 > MC = 32.55), writing (ME = 41.86 > MC = 39.68), meaning comprehension (ME = 55.83 > MC = 52.59), and cultural customs cognition (ME = 29.18 > MC = 27.94). Notably, overall scores (*p* = 0.000 < 0.05) and character recognition (*p* = 0.005 < 0.05) were particularly significant differences. These findings suggest that CUA-TCC is more effective than TSSA-TCC in enhancing students' literacy skills.

**Table 1:** Results of independent *t*-test on pre-test Chinese character achievement.

Group	*N*	Mean	SD	*t*	df	*p*
Experimental	38	81.3684	6.08685	−0.382	75	0.703
Control	39	81.9359	6.89792			

**Table 2:** Results of independent *t*-test on post-test Chinese character achievement.

Variable	Group	*N*	Mean	SD	*t*	df	*p*
Overall score	Experimental	38	91.9342	7.29881	4.222	75	0.000^***^
	Control	39	84.0256	9.02405			
Chinese character recognition	Experimental	38	34.1184	2.47538	2.881	75	0.005^**^
	Control	39	32.5513	2.29644			
Chinese character writing	Experimental	38	41.6842	3.56867	2.402	75	0.019^*^
	Control	39	39.6795	3.74954			
Chinese character semantic understanding	Experimental	38	55.8289	5.34015	2.105	75	0.039^*^
	Control	39	52.5897	7.88309			
Chinese character cultural cognition	Experimental	38	29.1842	2.41174	2.307	75	0.024^*^
	Control	39	27.9359	2.33728			

^*^p < 0.05, ^**^p < 0.01, ^***^p < 0.001.

### 4.2 The difference in learning motivation

Table 3 shows no significant difference in the pre-test motivation between groups (*t* = −1.061, *p* = 0.292 >0.05), suggesting that they exhibited comparable levels of motivation before the experiment. However, post-test results (Table 4) demonstrate that the experimental group outperformed the control group in overall learning motivation (ME = 27.5 > MC = 22.97, *p* = 0.001 < 0.05), intrinsic motivation (ME = 11.87 > MC = 9.49, *p* = 0.000 < 0.05), and extrinsic motivation (ME = 15.63 > MC = 13.49, *p* = 0.009 < 0.05). These findings indicate that the CUA-TCC significantly enhances students' learning motivation in both intrinsic and extrinsic dimensions of motivation.

**Table 3:** Results of Independent *t*-test on pre-test of learning motivation scores.

Variable	Group	*N*	Mean	SD	*t*	df	*p*
Learning motivation	Experimental	38	21.4474	6.25018	−1.061	75	0.292
	Control	39	22.9744	6.37217			

**Table 4:** Results of independent *t*-test on post-test learning motivation scores.

Variable	Group	*N*	Mean	SD	*t*	df	*p*
Learning motivation	Experimental	38	27.5000	4.94702	3.409	75	0.001^**^
	Control	39	22.9744	6.56741			
Intrinsic motivation	Experimental	38	11.8684	2.39562	3.930	75	0.000^***^
	Control	39	9.4872	2.89166			
Extrinsic motivation	Experimental	38	15.6316	2.87976	2.671	75	0.009^**^
	Control	39	13.4872	4.05146			

^**^p < 0.01, ^***^p < 0.001.

### 4.3 The difference in cognitive load

The results (Table 5) revealed that the experimental group's overall cognitive load (ME = 19.5 < MC = 22.76), and the sub-dimension of mental load (ME = 13.61 < MC = 14.38) and mental effort (ME = 5.89 < MC = 8.4) were all better than those of the control group. Moreover, the differences in overall cognitive load (*p* = 0.018 < 0.05) and mental effort (*p* = 0.000 < 0.05) were more significant between the two groups. However, it found no significant difference in mental load dimension (*p* = 0.462 > 0.05). This finding indicates that CUA-TCC effectively reduces students' cognitive load, particularly with regard to mental effort.

**Table 5:** Results of independent *t*-test on post-test cognitive load scores.

Variable	Group	*N*	Mean	SD	*t*	df	*p*
Cognitive load	Experimental	38	19.5000	5.47105	−2.426	75	0.018^*^
	Control	39	22.7692	6.30981			
Mental load	Experimental	38	13.6053	4.80731	−0.739	75	0.462
	Control	39	14.3846	4.44034			
Mental effort	Experimental	38	5.8947	2.25160	−4.195	75	0.000^***^
	Control	39	8.4103	2.95330			

^*^p < 0.05, ^***^p < 0.001.

### 4.4 Interview analysis

After the experiment, a 12–15-min semi-structured interviews were conducted with one teacher and six randomly selected students (three from each group) to explore their perceptions and experiences regarding the different teaching approaches (see Table 6).

**Table 6:** Interviewees' Information.

No.	Group	Codes in interview texts	Gender	Age
1	Experimental	E-S1	Female	9
2	Experimental	E-S2	Male	9
3	Experimental	E-S3	Female	8
4	Control	C-S4	Female	8
5	Control	C-S5	Female	9
6	Control	C-S6	Male	9
7	Teacher	T-7	Female	27

E = experimental group, C = control group, S = student, T = teacher.

#### 4.4.1 Qualitative data analysis based on word cloud diagram

**Figure 4:**
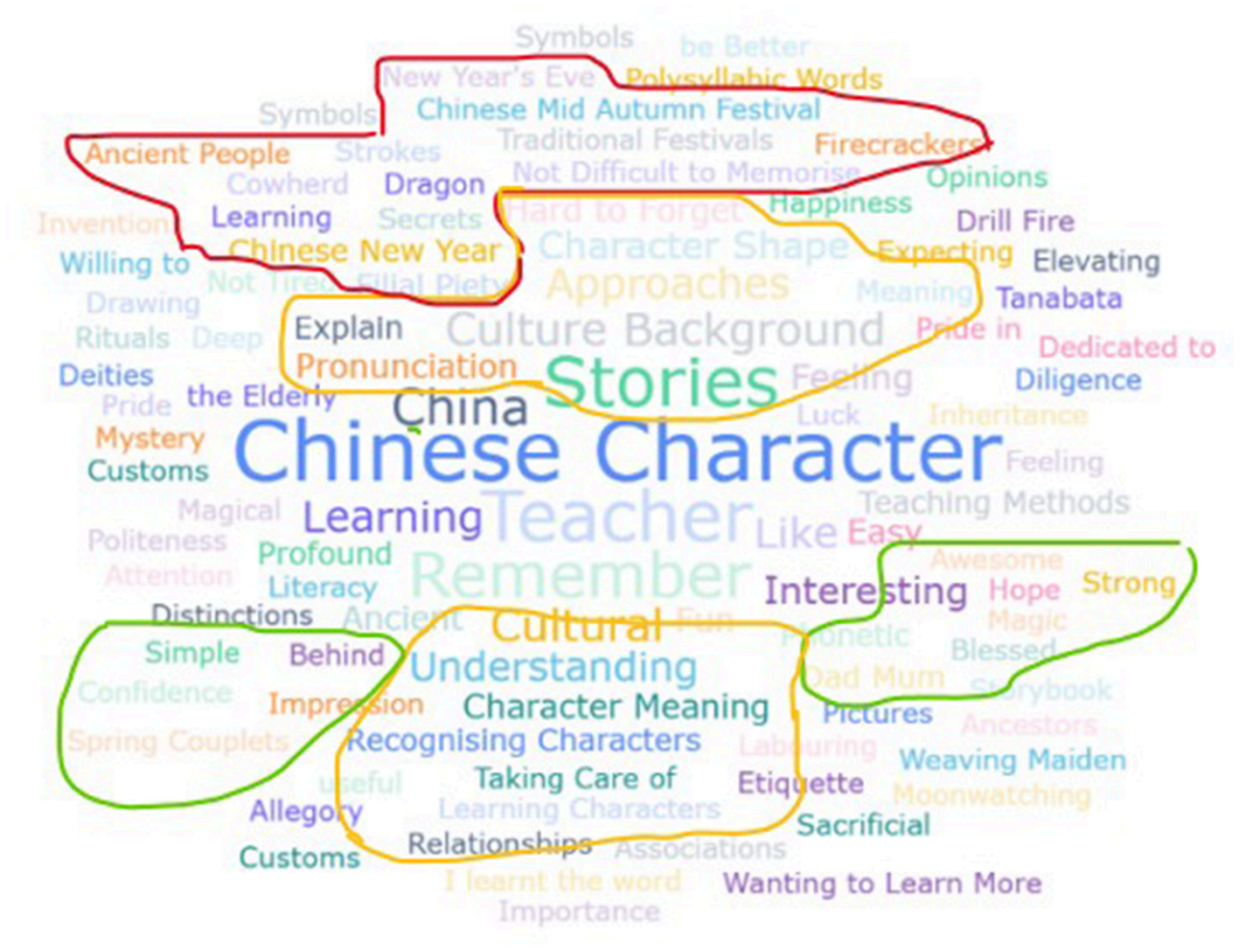
Word cloud of the experimental group.

**Figure 5:**
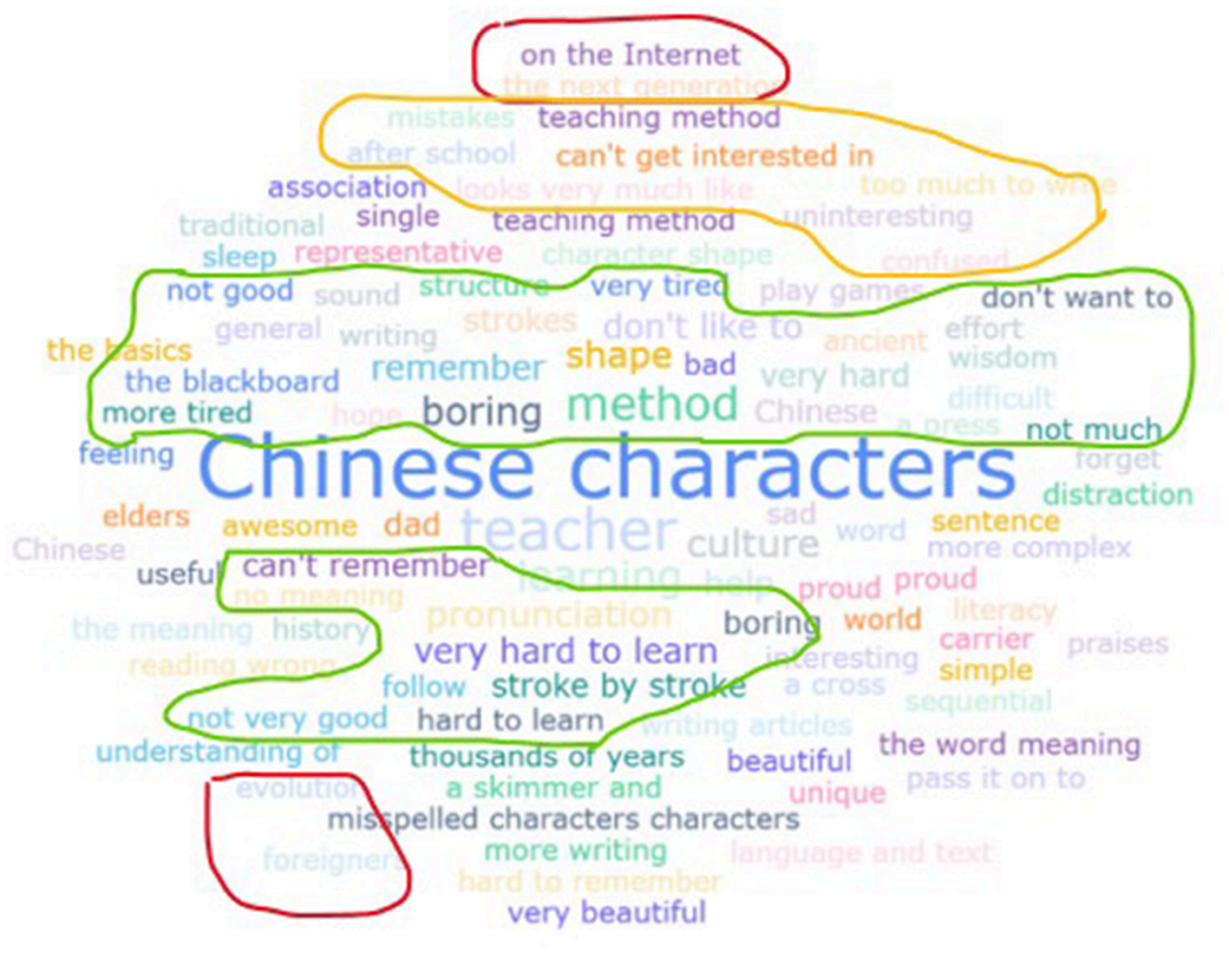
Word cloud of the control group.

This study extracted high-frequency keywords from qualitative data and generated a word cloud (Figures 4, 5) highlighting key differences between the two groups. The experimental group frequently mentioned “stories,” “remember,” and “cultural,” indicating enhanced engagement and cultural connection. Keywords like “explain,” “understand,” and “hard to forget” suggest improved memory strategies, while “tradition” and “festival” reinforce their cultural relevance. In contrast, the control group emphasized negative terms like “boring,” “cannot remember,” “confused,” and “make mistakes,” reflecting a rote-based approach. Additionally, words like “do not like,” “tired,” and “meaningless” used by students highlight the limitations of TSSA-TCC in fostering engagement and cultural identity.

#### 4.4.2 The qualitative data analysis

Grounded Theory (Charmaz, 2006) was applied to analyze the interview results through open, pivot, and core coding. Two researchers collaborated to ensure data reliability and consistency. Three main perspectives were distilled: learning performance, teaching approaches, and cultural understanding.

##### 4.4.2.1 Views on learning performance and teaching approaches

The interview results indicated that CUA-TCC improved students' memory of character forms and phonology while deepening their understanding through cultural stories. E-S3 shared, “I thought autumn (秋) was complex, but I can remember it after learning that 禾 represents crops and 火 symbolizes ripening crops.” This approach also helped students distinguish multi-pronounced characters. However, control group students struggled with pronunciation and orthographic recall, relying on rote memorization. C-S6 mentioned, “I often confuse whether 行 is pronounced háng or xíng. The teacher just repeats it for memorization.”

Experimental group students found CUA-TCC engaging and beneficial, as cultural stories reduced cognitive load, enhanced comprehension, and improved retention. E-S1 mentioned, “Memorizing strokes was boring, but stories make learning interesting and help me understand Chinese culture.” Teachers observed similar effects, as T-7 remarked: “Some disengaged students became attentive when hearing a story.” Conversely, control group students found TSSA-TCC dull and ineffective, as C-S6 expressed, “Learning Chinese characters is too hard and tiring. After writing for a while, I don't want to continue.”

##### 4.4.2.2 Views on cultural understanding

The interviews show that experimental group students better grasped the cultural significance of Chinese characters than the control group. First, CUA-TCC enhanced students' awareness of Chinese cultural imagery beyond orthographic and phonological aspects. E-S1 mentioned, “After hearing the story, I know that dragon (龙) symbolizes good fortune and strength, appearing in festivals like the New Year dragon dance.” The character 龙 was understood as a cultural symbol rather than just a word. This supports Takahashi's (2010) viewpoint that integrating cultural contexts promotes understanding and retention. In contrast, control group students focused on character forms and phonology. C-S4 said, “The teacher just asked us to remember shapes and pronunciations.”

CUA-TCC fostered emotional engagement, cultural interest and a preliminary understanding of the cultural connotations of Chinese characters. E-S2 noted, “After hearing ancient stories of filial piety (孝), I realized it is an important virtue. Now, I respect and help my parents with chores like sweeping the floor.” This illustrates how cultural narratives deepen emotional connections and transform abstract symbols into meaningful concepts (Christiana, 2013). E-S2 further stated, “中 is part of our country's name,” demonstrating how character learning enhanced students' attention to and emotional connection with Chinese culture. By linking personal values with cultural heritage, CUA-TCC strengthened students' cognitive understanding, emotional bonds, and social ties, thereby motivating long-term and active learning (Snow et al., 2020). In contrast, control group students lacked deeper cultural insight, expressing only general pride. C-S4 said, “My father told me Chinese characters are one of the oldest writing systems, which makes me proud.”

Moreover, CUA-TCC also fostered an awareness of the cultural transmission significance of Chinese characters. E-S3 shared, “Our teacher said 福 means good fortune, signifying good food, a warm home, and health. Now I understand why people hang up 福 during the New Year, and I will pass it on to my children.” This aligns with Heine (2007) and Markus (2016), who argued that cultural frameworks shape individual motivations and collective identities, fostering a sense of responsibility for cultural transmission. While control group students recognized inheritance intuitively, their understanding lacked depth. C-S5 said, “Our elders teach us Chinese characters. Then we pass them on.”

In conclusion, the interviews indicate that CUA-TCC enhances students' awareness of and interest in Chinese culture, promotes their understanding of the cultural significance of Chinese characters, and increases their appreciation of cultural traditions, addressing RQ4.

## 5 Discussion

### 5.1 CUA-TCC enhances Chinese character learning performance

For the discussion of RQ1, the results of this study reveal significant differences in Chinese character performance between the two groups. The experimental group demonstrated higher post-test scores in character recognition, writing, semantic understanding, and cultural cognition compared to the control group. This result is consistent with the studies of Cataldo and Alanís (2021), which emphasize that the integration of cultural elements can enhance learners' literacy skills. Notably, this study found that the incorporation of cultural elements had a particularly significant impact on the phonetic recognition of Chinese characters. As highlighted by Gillispie (2021), integrating culturally relevant stories and activities creates a more engaging learning environment, boosts learners' motivation and contextual understanding, and indirectly improves phonetic recognition. Qualitative interview data further confirmed that the integration of cultural content helped students establish multidimensional memory connections for mastering character forms, phonology, and semantics, as well as for overcoming the challenges of polyphonic characters.

Additionally, students in the control group commonly reported during interviews that their lack of interest in TSSA-TCC, led to lower engagement in learning Chinese characters. This finding corroborates Christiana's (2013) study on the limitations of TSSA-TCC. It further highlights the advantages of incorporating cultural context into teaching. CUA-TCC integrates the origins and usage scenarios of Chinese characters, and enables learners to establish emotional and cognitive connections with the material. This approach stimulates associative thinking and enhances the learning and retention of Chinese characters (Ghafar, 2024). It significantly improves learning efficiency, particularly in understanding and remembering the semantics of characters. Learning Chinese characters is not merely about memorizing orthographic forms and phonetics; it also involves understanding the culture and history embedded within them. The forms and semantics of Chinese characters often reflect aspects of ancient life, natural phenomena, and social values. Mastering these cultural norms helps learners better comprehend and retain the features of language (Takahashi, 2010).

### 5.2 CUA-TCC strengthens students' learning motivation

For RQ2, the findings indicate that the experimental group demonstrated significantly higher intrinsic motivation than the control group, suggesting that CUA-TCC effectively stimulates students' interest and initiative in learning. During interviews, students frequently mentioned “interesting” and “fun,” reflecting how cultural integration enriched their learning experience and emotional satisfaction, thereby boosting their intrinsic motivation. This aligns with Christiana (2013) and Oktarina et al. (2022), who found that embedding vivid historical and cultural meanings in instruction enhances student engagement and learning outcomes.

Additionally, cultural stories captured students' attention, fostered identity connections, and stimulated literacy motivation, consistent with Obidovna and Tokhirovna's (2024) study. In this study, through telling cultural stories (e.g., embodying filial piety, family harmony, etc.), students perceived their roles in the culture, generating intrinsic motivation aligned with the group's cultural identity. This supports Heine's (2007) and Markus's (2016) research that cultural contexts can shape motivation by presenting group goals and social values. In Asian collectivist cultures, such narratives foster students' sense of mission in cultural heritage, increasing engagement and learning motivation.

Furthermore, the experimental group showed significantly higher external motivation than the control group. Students frequently mentioned that cultural stories facilitated character retention, and sharing these stories with family members earned positive reinforcement, fostering a sense of achievement that further encouraged active learning. This aligns with Lo's (2024) view that cultural factors promote self-directed learning by stimulating students' interest and cultural understanding. External motivation was also evident in students' eagerness for continued cultural instruction, reinforcing Vansteenkiste et al. (2022) assertion that external motivation must be internalized for sustained learning engagement. This is a practical insight for teachers in instructional design.

In contrast, control group students found that the traditional method was monotonous, with repetitive memorization reducing engagement. This comparison further demonstrates the advantages of integrating culture into literacy instruction in promoting motivation and engagement.

### 5.3 CUA-TCC Reduces cognitive load in Chinese character learning

For RQ3, results showed that experimental group students had significantly lower mental effort than those in the control group. This indicates that students' active cognitive efforts, including cognitive efforts of perception, memory, thinking, and imagining, as well as emotional regulation and time control (Paas and Van Merrienboer, 1994), showed lower burden and higher efficiency after using the CUA-TCC. By providing contextual support through cultural stories, students were able to understand the meaning and usage of complex glyphs and multi-syllabic characters with less learning difficulty. Experimental group students recalled the orthographic-semantic associations of complex characters through stories, reflecting how cultural narratives provide multidimensional meanings and contexts (Bowman, 2018), enhancing overall cognitive engagement (Andi et al., 2024). Interview feedback further confirmed that emotional and cognitive connections fostered by cultural stories enhanced memory efficiency and long-term retention (Ghafar, 2024).

Conversely, control group students reported that TSSA-TCC increased learning pressure due to repetitive memorization and copying, leading to boredom and fatigue. This is consistent with Sweller's (1988) cognitive load theory, which suggests that CUA-TCC reduces the extrinsic load (e.g., eliminating students' comprehension barriers due to lack of context) and optimizes correlative load (e.g., introducing situational stories and increasing in-depth processing of the material). Teacher feedback further confirmed these advantages. Positive feedback from experimental group students regarding improved comprehension and retention of complex characters.

However, no significant difference was found in the mental load dimension, indicating that CUA-TCC did not lower students' mental load. According to Paas and Van Merrienboer (1994), the mental load is an interaction between the learning material complexity and the learner experience. A possible explanation for this result is self-regulation, which affects mental load performance and learning performance (Yan et al., 2023). While self-regulatory behaviors help allocate cognitive resources in moderately difficult tasks, they may increase cognitive load when tasks are too easy or difficult, leading to longer processing times and reduced learning efficiency (Blissett et al., 2018). Additionally, the integration of linguistic rules with cultural elements, along with the linguistic complexity of storytelling, may have required extra cognitive resources, increasing cognitive effort, slowing response times, and affecting comprehension (Cormier et al., 2014).

## 6 Conclusion and limitation

The CUA-TCC proposed in this study not only improved the learning performance of second-grade students but also enhanced their learning motivation and cultural understanding, and reduced cognitive load. Overall, this study provides valuable theoretical references and empirical support for innovative practices in elementary school Chinese character education and offers guidance for future research in Chinese character instruction.

Although this study yielded positive results, several limitations remain. First, the short duration of the experiment (7 weeks) likely reflects short-term progress, and developing students' literacy skills usually requires a longer period of practice and feedback (Ryan and Dörnyei, 2013). Therefore, the long-term effects of the CUA-TCC remain unclear. Second, the sample size, is limited to second-grade students, which may affect the generalizability of the results. Additionally, the complexity of learning materials or the language complexity used by teachers when narrating cultural stories may have hindered the reduction in cognitive load, potentially impacting the effectiveness of the CUA-TCC.

To address these issues, future studies could extend the duration of the experimental period (e.g., 2–3 months) to verify the long-term effectiveness of the CUA-TCC, expand the sample size and explore the applicability of the CUA-TCC across different grade levels, and incorporate richer data collection methods such as classroom observations and long-term follow-ups. Furthermore, future research should explore how to balance teaching effectiveness and cognitive load when designing cultural story content.

## Data Availability

The raw data supporting the conclusions of this article will be made available by the authors, without undue reservation.
