# Supplementary material for: The impact of cultural understanding-based approach to teaching Chinese characters on primary school students' performance, motivation, cognitive load, and culture understanding
Source: Front Psychol. 2026 Jul 21;17:1839204. doi: 10.3389/fpsyg.2026.1839204 (PMC13433743; doi:10.3389/fpsyg.2026.1839204)
Supplement: Supplementary file 1 [file Supplementary_file_4.pdf]

Assessment paper on language learning in Year 2 of primary school

小学二年级语文学习情况评估卷

Time: 60 minutes　　100 full marks

时间：60 分钟　满分 100 分

Name 姓名： ______________ **Class 班级：** ______________ **Mark 成绩：** ______________

I. Look at the picture and guess what word should be filled in the grid blank? （3 points）

一、看图猜一猜，田字格里应该填什么字？（3 分）

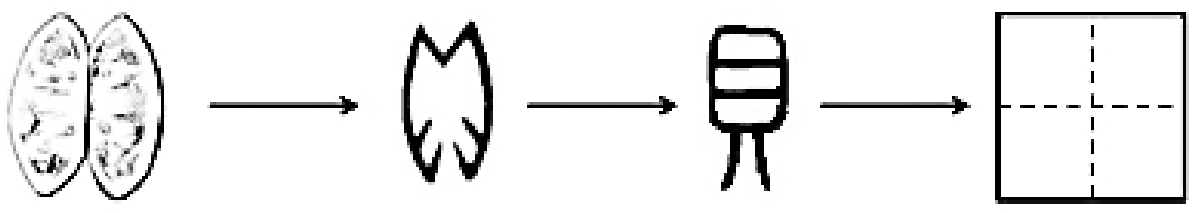

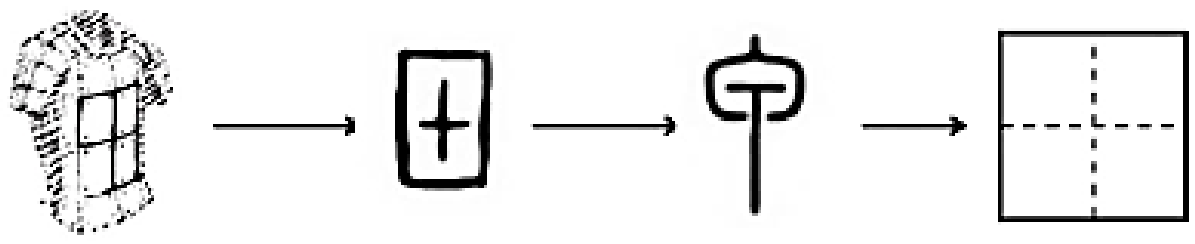

II. Read the sentences and write the words according to Pinyin (14 points)

二、读读句子，根据拼音写词语 （14 分）

zhuǎn yǎn（　　）又是新春到，全家 tuán yuán（　　）真 rè nào（　　）。

桌 上 měi shí（　　）真不少，kǎo yā（　　）、羊肉和蒸饺，还有 hóng shāo（　　）qié zi（　　）煲。

III. Choose the correct pronunciation for the dotted polyphonic words in the following two tasks.（10 points）

三、给下列两个任务中的加点的多音字选择正确的读音。(10 分)

Task 1

任务一：

①piāo ② piào

1.落叶随着溪水，漂(　　　)流到远方。

2.妈妈给我买了一件漂(　　　)亮的新衣服。

① zhá ② zhà

3.这家店的炸(　　)鸡很有名，有机会我一定要品尝一下。

4.烟花在空中炸(　　　)开，如同仙女散花一般。

Task 2

任务二：

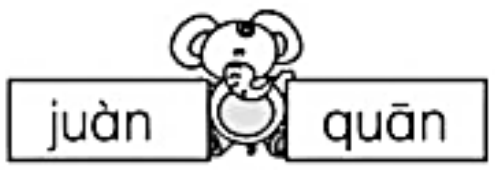

羊圈（　　　　）

一圈（　　　　）

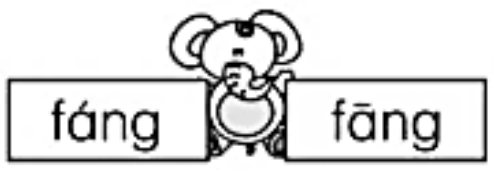

街坊（　　　　）

磨坊（　　　　）

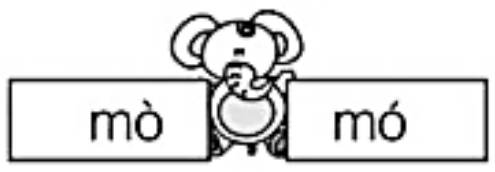

磨刀（　　　　）

石磨（　　　　）

IV. Choose the correct option and fill in the brackets.（8 points）

四、选择正确的选项填到括号里。(8 分)

1.One of the following without typos is （　　）

1.下列没有错别字的一项是 （　　）

A.广洲　　　B.台弯　　　C. 海峡

2. The Chinese characters to be inserted in the following lines are (　　)

2. 填入以下横线的字分别是 （　　）

有水把茶______，有足快快_______，有饭能吃______,有火放鞭________。

A.饱、泡、袍、炮　　B. 泡、跑、饱、炮　　C.饱、炮、泡、跑

3. Which of the following words has nothing to do with "mood, emotion"? (   )

3.下面哪个字表示的意思与"心情、情感"无关？ （　　）

A. 悔　　　　　　B.思　　　　　　C. 分

4. Grandma wants to eat lighter cuisine for lunch. Would you recommend it to her ( ).

4.奶奶午饭想吃清淡一点的菜系，你会为她推荐（　　　）。

A.葱爆羊肉　　　　B.炸酱面　　　　C.清蒸鲈鱼

V. Follow the example and do it. （4 points）

五、照样子，做一做。(4 分)

例：该-讠 ＋ 刂→刻　刻画

疲 - _____ + _____ → _____ （　　　　）

说- _____ + _____ → _____ （　　　　）

VI. Take your pick and replace the part of the sentence drawn "______" with an idiom. （6 points）

六、选一选，把句子中加粗并画"______"的部分换成成语。(6 分)

筋疲力尽　揠苗助长　自言自语

（1）丁丁**自己对自己说**："要是有个朋友陪我一起上学多好啊！" （　　　　　　）

（2）小力才三岁就报了五种课外班，这真是**拔起禾苗帮他长**啊。（　　　　　　）

（3）我们用了三个小时才爬到山顶，累得**一点儿力气也没有了**。（　　　　　　）

VII. Fill in the table as required by the dictionary. (12 points)

七、按查字典的要求填表。(12 分)

要查的字	部首	除部首外的笔画数	音序	音节	结构	组词
烤						
热						

财						
茄						

VIII. Write sentences as required. (10 points)

八、按要求写句子。（10 分）

1.例：小柳树的腰细细的，树枝绿绿的，真好看。（照样子，补充句子）

苹果的颜色__________________，味道______________________，真好吃。

2. 古时候，人们觉得贝壳很漂亮，很珍贵，喜欢把他们当作饰品戴在身上。（任选一个加下划线的词语，仿写句子）

____

3. 我会连词成句，并加上标点。

小马　跑去　飞快地　磨坊　往

____

4. 十二生肖我知道 （4 分）

我的生肖是______。我的朋友小明的生肖是卯_______。

小明哥哥比他大 2 岁，他哥哥的生肖是_______;

小明弟弟比他小 1 岁，他弟弟的生肖是_______。

IX. Judge whether the following sentences are correct or not. Mark √ if they are correct and × if they are incorrect. (3 points)

九、根据所学内容，判断下列句子说法是否正确，正确的打√，错误的打×。（3 分）

(1) 做什么事都要自己亲自尝试，根本不能听信别人的话。(　)

(2) 看问题做事情要从客观存在的实际出发，实事求是，不要轻易下结论。(　)

(3) 解决问题要遵循事物的规律，不能急于求成。(　)

X. Arrange the following jumbled sentences into a coherent paragraph and put the sequence number in the brackets. (5 points)

十、把下列错乱的句子排成一段通顺的话，将序号填在括号里。（5 分）

(　　)它使我知道，不要拿自己的长处去比别人的短(duǎn)处，要学习别人的长处。

(　　)它使我知道，当发现了错误，只要及时改正还不算晚。

(　　)我喜欢《小柳树和小枣树》这个故事。

(　　)我常把这两个故事讲给朋友听，

(　　)我也喜欢《我要的是葫芦》这个故事,

XI.Theme Exploration. (6 points)

十一、主题探究。(6 分)

我的理想。

小明:我长大以后想当一名工程师。

小刚:为什么呢?

小明:因为____________________________________。

小刚:我长大以后想__________________________________。

小明:说说你的理由。

小刚:我要________________________________。

XII. Extracurricular reading. (9 points)

十二、课外阅读。(9 分)

饺子

饺子是一种面食，用面粉做成薄饼，裹(guǒ)着馅，一般捏成弯月的形状。饺子的历史可长了,它起源于东汉时期。最早的饺子混着汤一起吃,吃法像现在的馄饨(hún tun)。到了唐代，饺子就已经和今天的一模一样了。

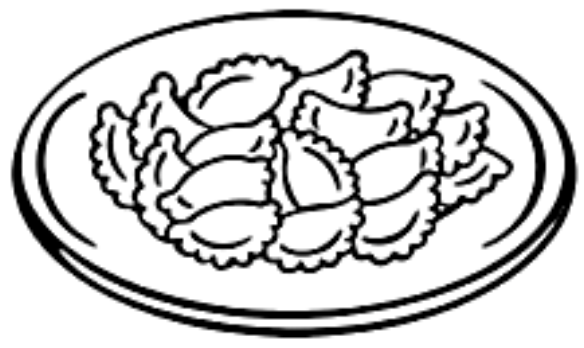

北方人过年有吃饺子的习俗，为什么春节要吃饺子呢? 这里面有很多民间传说。有的说是为了纪念盘古开天辟地，结束了天地间的混沌状态; 有的说饺子的形状像元宝，吃饺子是为了“招财进宝”。不管是什么样的传说，都寄托了人们对美好生活的向往。

春节吃饺子有很多讲究。除夕的晚上，一家人热热闹闹地一起动手包饺子，要在午夜前包好。等到午夜零点，钟声敲响，在这辞旧迎新的时刻,全家人就可以围着桌子吃饺子。有的人家做饺子时还会在饺子里偷偷藏一枚钱币或一颗栗(lì)子，寓意谁吃到了谁就会交好运。

1.本文一共有______个自然段。

2.饺子起源于什么时期? (　　　　)

A. 唐代

B.东汉

C.清代

3.下列说法错误的是 (　　　　)。

A.到了唐代，饺子就是今天的样子了。

B.最早的饺子是混着汤一起吃。

C.有的人家做饺子时还会偷偷藏一个糖果在里面，寓意谁吃到了谁就会交好运。

4.春节吃饺子有什么寓意?从文中找出答案，用“________”画出来。

5.饺子怎么做呢?你能简单说一说吗?

____

____

____

XIII. Writing (points)

十三、写作（10）

看图想一想，他们想要干什么？再发挥想象，试着把故事补充完整再写下来。(不会写的字可以写拼音)

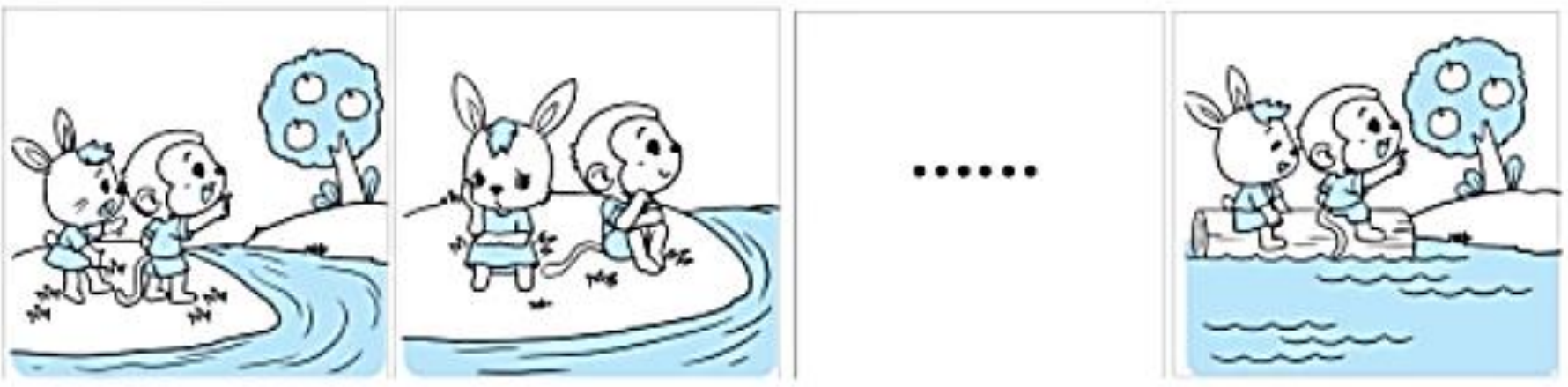

____

____

____

____

____

____

____
