# Supplementary material for: The impact of cultural understanding-based approach to teaching Chinese characters on primary school students' performance, motivation, cognitive load, and culture understanding
Source: Front Psychol. 2026 Jul 21;17:1839204. doi: 10.3389/fpsyg.2026.1839204 (PMC13433743; doi:10.3389/fpsyg.2026.1839204)
Supplement: Supplementary file 2 [file Supplementary_file_1.docx]

Supplementary Appendix A Learning Motivation Scale

# 1 Intrinsic motivation

1. I think learning Chinese characters is not only interesting but also valuable.

2. I think the learning of Chinese characters in the language programme is well worth knowing more about.

3. I will actively look for more information in order to further my knowledge of Chinese characters.

# 2 Extrinsic motivation

4. I hope I can learn more about Chinese characters in Chinese language course and improve my performance.

5. It is very important for me to master Chinese characters.

6. It is important and meaningful for me to learn about Chinese characters related to our life.

7. I think everyone should pay attention to and learn Chinese characters.
