# Supplementary material for: The impact of cultural understanding-based approach to teaching Chinese characters on primary school students' performance, motivation, cognitive load, and culture understanding
Source: Front Psychol. 2026 Jul 21;17:1839204. doi: 10.3389/fpsyg.2026.1839204 (PMC13433743; doi:10.3389/fpsyg.2026.1839204)
Supplement: Supplementary file 3 [file Supplementary_file_2.docx]

Supplementary Appendix B Cognitive Load Scale

# 1 Mental Load

1. The learning content in this learning activity is difficult for me.

2. I have to work very hard to answer the questions in this learning activity.

3. I find it troublesome to answer the questions in this learning activity.

4. I feel frustrated when answering the questions in this learning activity.

5. I don't have enough time to answer the questions in this learning activity.

# 2 Mental Effort

6. The way the lesson was delivered or the learning content was presented in the learning activity required a great deal of mental effort on my part.

7. I need to put in a lot of effort to complete the learning tasks or meet the learning objectives in this learning activity.

8. The teaching style in the learning activity is difficult to follow and understand.
