# Supplementary material for: The impact of cultural understanding-based approach to teaching Chinese characters on primary school students' performance, motivation, cognitive load, and culture understanding
Source: Front Psychol. 2026 Jul 21;17:1839204. doi: 10.3389/fpsyg.2026.1839204 (PMC13433743; doi:10.3389/fpsyg.2026.1839204)
Supplement: Supplementary file 4 [file Supplementary_file_3.docx]

Supplementary Appendix C Interview Protocol

# 1 I. Improvement of learning motivation

1. Do you feel more interested in learning Chinese characters with cultural background? Why?

2. Which literacy method do you prefer? How did the stories with cultural background affect your learning?

3. Do you think this teaching method makes you more willing to learn Chinese characters? Are there any examples to illustrate this?

# 2 II. Reduction of cognitive load

1. Do you think it is easier for you to remember Chinese characters when you learn Chinese characters with cultural backgrounds? Why?

2. Do you think this method has helped you learn more easily when understanding the shape, sound and meaning of the characters?

3. Do you find it easier to learn Chinese characters in this way than in other ways?

# 3 III. Impact on Learning Achievement (pronunciation, forms, meaning)

1. Do you feel that you understand the meanings of the characters better than before after learning the cultural background of the characters? Can you give me some examples?

2. Have you encountered any difficulties in understanding the pronunciation, shape, and meaning of Chinese characters? Did this method help you solve them?

3. Did the cultural background help you remember the meaning of the characters more easily? Do you have any examples of impressive Chinese characters?

# 4 IV. Views on Cultural understanding

1. Do you think that learning the cultural stories of Chinese characters has helped you understand more about Chinese culture? Can you give examples?

2. Do you think that learning Chinese characters with cultural backgrounds makes you feel proud of your own culture?

3. Has this teaching method made you more interested in Chinese characters and Chinese culture?
