# Supplementary material for: The impact of cultural understanding-based approach to teaching Chinese characters on primary school students' performance, motivation, cognitive load, and culture understanding
Source: Front Psychol. 2026 Jul 21;17:1839204. doi: 10.3389/fpsyg.2026.1839204 (PMC13433743; doi:10.3389/fpsyg.2026.1839204)
Supplement: Supplementary file 5 [file Supplementary_file_5.docx]

***Supplementary Appendix E***

**Table of Specification of Content for Test Items**

| Learning Objectives | Chinese Character Learning Performance | | | |
| --- | --- | --- | --- | --- |
|  | **Recognition** | **Writing** | **Semantic Understanding** | **Cultural Cognition** |
| I. Look at the picture and guess what word should be filled in the grid blank? |  | / |  |  |
| II. Read the sentences and write the words according to Pinyin. | / | / |  | / |
| III. Choose the correct pronunciation for the dotted polyphonic words in the following two tasks. | / |  |  |  |
| IV. Choose the correct option and fill in the brackets |  | | | |
| 1.One of the following without typos is ( ). |  | / |  |  |
| 2.The Chinese characters to be inserted in the following lines are ( ). |  |  | / |  |
| 3. Which of the following words has nothing to do with "mood, emotion"? ( ) |  |  | / |  |
| 4. Grandma wants to eat lighter cuisine for lunch. Would you recommend it to her ( ). |  |  | / | / |
| V. Follow the example and do it. |  | / |  |  |
| VI. Take your pick and replace the part of the sentence drawn "______" with an idiom. |  |  | / |  |
| VII. Fill in the table as required by the dictionary. | / |  | / |  |
| VIII. Write sentences as required. | | | | |
| VIII 1 |  | / | / |  |
| VIII 2 |  | / | / | / |
| VIII 3 |  | / | / |  |
| VIII 4 |  | / | / | / |
| IX. Judge whether the following sentences are correct or not. Mark √ if they are correct and × if they are incorrect. |  |  | / |  |
| X. Arrange the following jumbled sentences into a coherent paragraph and put the sequence number in the brackets. |  |  | / |  |
| XI.Theme Exploration. |  | / | / |  |
| XII. Reading and Comprehension. | | | | |
| XII 1 |  |  | / | / |
| XII 2 |  |  | / | / |
| XII 3 |  |  | / | / |
| XII 4 |  |  | / | / |
| XII 5 |  |  | / | / |
| XIII. Writing |  | / | / |  |
